# Improved Immune Moth–Flame Optimization Based on Gene Correction for Automatic Reverse Parking

**DOI:** 10.3390/s24072270

**Published:** 2024-04-02

**Authors:** Gang Liu, Xinli Xu, Longda Wang

**Affiliations:** 1 College of Engineering, Inner Mongolia Minzu University, Tongliao 028000, China; liu_gang530242@163.com; 2 Department of Automation, Shanghai Jiao Tong University, Shanghai 200240, China; 3 School of Mechanical Engineering, University of Shanghai for Science and Technology, Shanghai 200093, China; xuxinli20061013@163.com; 4 School of Automation and Electrical Engineering, Dalian Jiaotong University, Dalian 116026, China

**Keywords:** automatic reverse parking, immune moth–flame optimization, reference trajectory optimization, adaptive inertia weight coefficient, parameter optimization, gene correction

## Abstract

During the process of reverse parking, it is difficult to achieve the ideal reference trajectory while avoiding collision. In this study, with the aim of establishing reference trajectory optimization for automatic reverse parking that smooths and shortens the trajectory length and ensures the berthing inclination angle is small enough, an improved immune moth–flame optimization method based on gene correction is proposed. Specifically, based on the standard automatic parking plane system, a reasonable high-quality reference trajectory optimization model for automatic parking is constructed by combining the cubic spline-fitting method and a boundary-crossing solution based on gene correction integrated into moth–flame optimization. To enhance the model’s global optimization performance, nonlinear decline strategies, including crossover and variation probability and weight coefficient, and a high-quality solution-set maintenance mechanism based on fusion distance are also designed. Taking garage No.160 of the Dalian Shell Museum located in Dalian, Xinghai Square, as the experimental site, experiments on automatic parking reference trajectory optimization and tracking control were carried out. The results show that the proposed optimization algorithm provides higher accuracy for reference trajectory optimization and can achieve better tracking control of the reference trajectory.

## 1. Introduction

Automatic parking behavior whereby vehicles meet the reverse parking conditions from a starting area within an allowable range is called automatic reverse parking. With the rapid development of transportation, the demand for comprehensive performance indicators for intelligent vehicles is increasing, and the performance quality of intelligent ARP functions is also increasingly scrutinized [1]. The reverse automatic parking system is indispensable for intelligent vehicles to ensure optimal reverse parking performance indicators, such as safety, comfort, speed, and accuracy. Its reference trajectory optimizer should exhibit reverse parking trajectory optimization performance to provide collision-free, accurate automatic parking trajectories, and the path length should be smooth and short and the berthing inclination angle small [2,3]. However, given the optimization abilities of existing optimization algorithms, the ideal conditions are difficult to achieve [4].

Regarding the problems encountered when applying automatic parking systems to intelligent vehicles, scholars have carried out a series of studies. As early as 1996, a novel neural network architecture for trajectory generation and the control of automated car parking was proposed [5]. In [6], the authors undertook the design, testing, and validation of a deep neural network (DNN)-based control scheme for autonomous ground vehicles (AGVs) during the parking process. An automatic vacant parking space management system using multicamera vehicle detection was presented in [7]. A graph-based algorithm for the optimal control of switched systems for application to car parking was developed in [8]. A distributed control method for coordinating multiple vehicles within the framework of an automated valet parking system was introduced in [9]. However, there are relatively few effective improvement strategies for the reference trajectory optimization of intelligent ARP.

The parking trajectory optimizer has a strong influence on the performance quality of automatic parking systems. In recent years, intelligent optimization algorithms have received significant attention from researchers in the field of automatic parking, with many scholars around the world having conducted studies on this topic. A computational trajectory optimization framework for multiobjective automatic parking motion planning was proposed in [10]. An improved A* and dynamic window approach was proposed in [11] to enhance the accuracy and speed of automatic parking in tracked vehicles. Improved ant colony optimization (ACO) for path planning in an AGV-based intelligent parking system was introduced in [12]. Improved immune moth–flame optimization (IIMFO) based on the immune mechanism, Gaussian mutation mechanism, and opposition-based learning strategy was proposed for automatic parking path optimization in intelligent vehicles in [13]. However, the high-performance optimization frameworks using improved immune moth–flame optimization based on gene correction (IIMFO-GC) for ARP trajectory optimization lack guidelines for their design, so it may be difficult to obtain optimal ARP reference trajectories.

With the rapid development of the public transportation system, the quality requirements for daily travel are also increasing. However, for intelligent ARP, the reference trajectory optimization performance of traditional parking trajectory optimizers is often unsatisfactory. Based on a study [13] about reference trajectory optimization for automatic reverse parking in intelligent vehicles, the major contributions of this work are as follows:

(I) A reasonable high-quality reference trajectory optimization model for ARP is constructed by combining the cubic spline-fitting method and a boundary-crossing solution based on gene correction integrated into moth–flame optimization.

(II) An improved immune moth–flame optimization method is designed. Specifically, nonlinear decline strategies for weight coefficient and crossover and mutation probabilities, and a high-quality solution-set maintenance mechanism based on fusion distance, are designed to enhance the global optimization performance of immune moth–flame optimization.

(III) A gene correction method is designed to establish a highly effective boundary-crossing solution for ARP; on this basis, an IIMFO-GC is proposed and used in the trajectory optimizer of the ARP system to further improve reference trajectory optimization performance.

This paper is organized as follows: Section 2 introduces the reference trajectory optimization model for ARP; Section 3 introduces the gene correction method for reverse parking; Section 4 introduces the IIMFO; Section 5 presents the ARP experimental results and simulation analysis; and Section 6 concludes the article.

## 2. Reference Trajectory Optimization Model for ARP

### 2.1. Basic Principles of ARP

**Figure 1:**
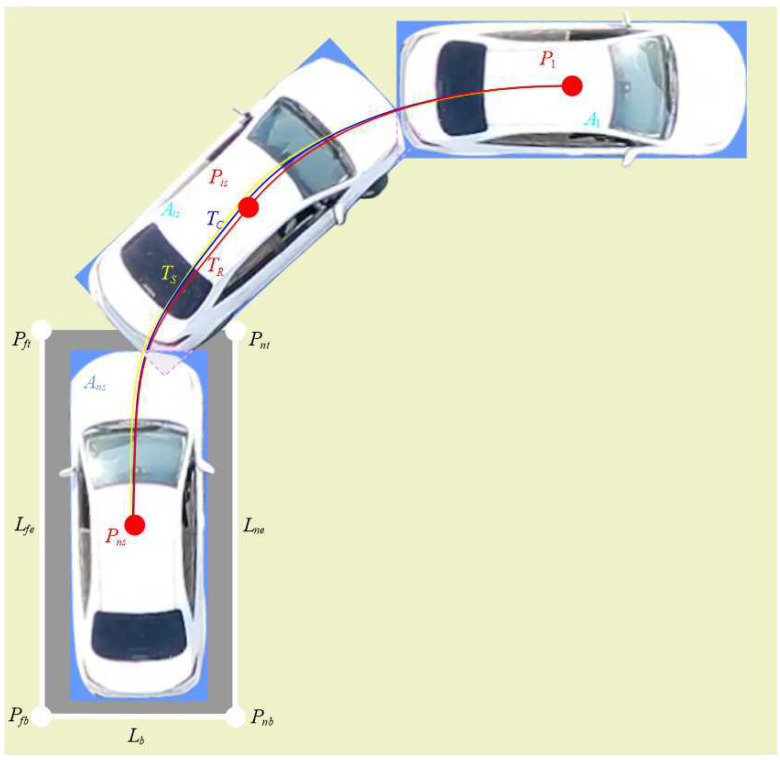
Schematic diagram of the ARP principle.

ARP generally adopts the method of automatic reversing. A schematic diagram of the ARP principle is shown in Figure 1.

In Figure 1, it is assumed that the vehicle coverage areas are purposefully collected in sequence ns times in the ARP process, which is denoted as the collected vehicle coverage area set $\left(A_{1},\cdots ,A_{is},\cdots ,A_{ns}\right)$. In this figure, $A_{1}$ represents the parking starting area, $A_{ns}$ is the parking stop area, and $P_{1}$, $P_{is}$, and $P_{ns}$ are the center points of the collected vehicle coverage areas. $L_{d}$ represents the bottom line of parking garage, and $L_{ne}$ and $L_{fe}$ are the near and far side lines of the parking garage, respectively. $P_{nt}$, $P_{ft}$, $P_{nb}$ and $P_{fb}$ are the near and far corner points of the garage top and the near and far corner points of garage bottom, respectively. $T_{R}$ and $T_{C}$ are the reference parking trajectory and the actual tracking control trajectory, respectively. Due to the uncertain interference in the parking process, it is impossible to achieve a tracking control error of zero, that is, $T_{C}$ cannot completely coincide with $T_{R}$. $T_{S}$ is the tracking control trajectory monitoring value obtained by the sensor and image system. Due to the limitation in sensor accuracy and measurement error, it is impossible to achieve an error value of zero for tracking control trajectory monitoring, that is, $T_{S}$ cannot completely coincide with $T_{C}$.

### 2.2. Constraint Judgment of ARP Feasibility

ARP usually requires that there are no obstacles in the parking environment, that the parking garage is known, and that the bottom and side lines of the parking garage, as well as the bottom and top corners of the parking garage, are clearly identifiable. In addition, the constraint on the starting feasibility of parking in the garage should be satisfied: the center point $P_{1}$ of the starting area should be within the feasible starting area $\Omega_{C,1}$. The angle between the line connecting the center point $P_{1}$ of the starting area with its bottom midpoint $P_{b}1$ and the bottom line $L_{d}$ of the garage is called the starting inclination angle $\Delta {∠}_{B}$. If its absolute value is less than the threshold $\Delta {∠}_{B\max}$, the constraint expression of the starting feasibility of reverse parking is as follows :

(1) $$\begin{array}{c} \begin{array}{c} \forall P_{1}\in \Omega_{C,1}\\ \left|{∠}_{B}\right|=\left|∠\left(T\left(P_{1},P_{b1}\right),L_{d}\right)\right|<\Delta {{∠}_{B}}_{\max} \end{array} \end{array}$$

### 2.3. Conversion Principle of ARP Plane Coordinate System

The standard ARP plane coordinate system takes the far corner point of the bottom of the garage as the origin and the bottom line of the garage as the positive horizontal axis (x-axis). Adopting a standard ARP plane coordinate system is not only beneficial for simplifying reverse parking calculations, but also enables drivers to evaluate real-time conditions and make more rational parking decisions. To convert the real scene’s ARP plane coordinate system obtained by the parking image system into a standard ARP plane system, we must carry out three steps: first, determine the largest range of the real rectangular parking scene Ω; then, rotate the coordinate system according to the real scene’s rotation angle ∠α; finally, translate the origin according to the coordinate $\left(x_{0},y_{0}\right)$ of the far corner point of the garage bottom in the real scene’s coordinate system.

Assume there is a point *s* in the parking area whose coordinate in the standard ARP plane system is $\left(x_{s},y_{s}\right)$ and whose angle with the horizontal axis is ∠β. Then, $∠\beta =\arcsin\left(\frac{y_{s}}{\sqrt{{x_{s}}^{2}+{y_{s}}^{2}}}\right)$.

If the coordinate $\left(x_{s},y_{s}\right)$ is known, the conversion formula for the coordinate $\left(x_{r},y_{r}\right)$ of the real scene’s reverse parking plane system can be obtained as follows:

(2) $$\begin{array}{c} \begin{array}{c} x_{r}=\sqrt{{x_{s}}^{2}+{y_{s}}^{2}}\cdot \cos\left(∠\beta -∠\alpha \right)+x_{0}\\ y_{r}=\sqrt{{x_{s}}^{2}+{y_{s}}^{2}}\cdot \sin\left(∠\beta -∠\alpha \right)+y_{0} \end{array} \end{array}$$

Similarly, if the coordinate $\left(x_{r},y_{r}\right)$ is known, it is easy to establish the coordinate $\left(x_{s},y_{s}\right)$.

In the standard parking coordinate system, the set of decision variables is set as ${\left\{\begin{array}{c} \left(x\left(P_{2}\right),y\left(P_{2}\right)\right)\\ ,\cdots \cdots ,\\ \left(x\left(P_{ns}\right),y\left(P_{ns}\right)\right) \end{array}\right\}}^{T}$, and $x\left(P_{is}\right)$ and $y\left(P_{is}\right)$ are the abscissa and ordinates of the central point $P_{is}$ of the is collected vehicle coverage areas in the standard reverse parking coordinate system. On the premise that the starting inclination angle $\Delta {∠}_{B}$ is small enough (${∠}_{B}\leq \Delta {∠}_{B\max}$), to obtain the reverse parking reference trajectory with the shortest length, it is known from the parking experience that the abscissa and ordinates about the center points of the collected vehicle coverage areas should decrease one by one. The expression is as follows:

(3) $$\begin{array}{c} x\left(P_{ns}\right)\leq ,\cdots ,\leq x\left(P_{is}\right)\leq ,\cdots ,\leq x\left(P_{1}\right) \end{array}$$

(4) $$\begin{array}{c} y\left(P_{ns}\right)\leq ,\cdots ,\leq y\left(P_{is}\right)\leq ,\cdots ,\leq y\left(P_{1}\right) \end{array}$$

### 2.4. Optimization Model for ARP Reference Trajectory

The number of center points of the collected vehicle coverage areas is set to ns, and the ARP reference trajectory can be obtained by fitting ns center points $P_{1},P_{2},\cdots ,P_{ns}$. Among them, the center point $P_{1}$ of the starting area is fixed, and the center points $P_{2},\cdots ,P_{ns}$ of the other n−1 collected vehicle coverage areas can be adjusted within a certain region. The periodic time of the ARP process is set to Δt, and the optimization model of the ARP reference trajectory is expressed as follows:

(5) $$\begin{array}{c} \begin{array}{c} \min\left\{\begin{array}{c} L\left(T_{R}\right)=\int_{0}^{T_{\max}}v(t)dt\approx \sum_{it=1}^{nt}\Delta L_{it}\\ \left|\Delta {∠}_{S}\right|=\left|∠\left(T\left(P_{ns},P_{bm}\right),L_{fe}\right)\right| \end{array}\right.\\ S.T.\left\{\begin{array}{c} T_{R}=tf\left(P_{1},P_{2},\cdots ,P_{ns}\right)\\ \Delta L_{it}\leq \Delta L_{\max}\\ \Delta {∠}_{it}\leq \Delta {∠}_{\max}\\ \forall P_{is}\in \Omega_{C,is}\\ \forall P_{g}\left(ig\right)\notin \Omega_{c,it}\\ \forall P_{g}\left(ig\right)\in \Omega_{g}\\ \left|\Delta {∠}_{S}\right|=\leq \Delta {{∠}_{S}}_{\max}\\ \left|\Delta y_{ns}\right|=\left|y\left(P_{ns}\right)-y_{H}\right|\leq \Delta {y_{H}}_{\max} \end{array}\right. \end{array} \end{array}$$

where $\Omega_{g}$ is the coverage area of the edges on both sides of the garage, $P_{g}$ is the collection of detected edge collision avoidance point positions, $n_{g}$ is the detected point position, $P_{g}\left(ig\right)$ is the ig-th detected edge collision avoidance point position, $i\in \left\{1,2,\cdots ,ng\right\}$ (then, $\forall P_{g}\left(ig\right)\in \Omega_{g}$), and $\Delta L_{it}$, $\Delta {∠}_{it}$, and $\Omega_{c,it}$ are the parking interval distance, attitude angle difference, and vehicle coverage area for the it-th period, respectively. In this study, the vehicle coverage area $\Omega_{c,it}$ is equalized as a rectangle with the length and width of the vehicle, $\Delta L_{\max}$ and $\Delta {∠}_{\max}$ are the threshold values for parking interval distance and attitude angle difference, $T_{\max}$ is the parking time, nt is the number of periods, $nt=\left\lceil \frac{T_{\max}}{\Delta t}\right\rceil$, $it\in \left\{1,2,\cdots ,nt\right\}$, ⌈⌉ is the rounding up operator, since the vehicle cannot collide with the bottom line of the garage and the edges on both sides, $\forall P_{g}\left(i\right)\notin \Omega_{c,it}$, $P_{is}$ is the center point of the is-th collected vehicle coverage area, $is\in \left\{2,\cdots ,ns\right\}$, $\Omega_{C,is}$ represents its adjustable region (then, $\forall P_{is}\in \Omega_{C,is}$), and tf is the reverse parking reference trajectory obtained through fitting, with the cubic spline-fitting method adopted in this study; $\Delta {∠}_{S}$ is the parking inclination angle, $\Delta {∠}_{S}=∠\left(T\left(P_{ns},P_{bm}\right),L_{fe}\right)$ and is the angle between the line $T\left(P_{ns},P_{bm}\right)$ connecting the center point $P_{ns}$ of the parking area with the midpoint $P_{bm}$ at the bottom of the garage and the far side line $L_{fe}$ of the garage, and $\Delta {∠}_{S\max}$ is the threshold for its absolute value; $\Delta y_{ns}$ is parking position error and is the error between the ordinate $y\left(P_{ns}\right)$ of the center point $P_{ns}$ of the parking area and the expected ordinate $y_{H}$, $\Delta {y_{H}}_{\max}$ is the threshold, and $T_{R}$ is the reference trajectory.

The cubic spline-fitting method is a spline curve-fitting method using the cubic spline interpolation function, and it has been widely used in various industrial applications. Assuming that the closed interval $\Delta =\left[a,b\right]$ and n+1 interpolation points $\left(x_{0},x_{1},\cdots ,x_{n}\right)$ within the interval Δ are known, in terms of the cubic spline interpolation function s(x), there are two kinds of features.

Firstly, regarding the arbitrary subinterval $\left[x_{i},x_{i+1}\right]$, $\left(i\in [0,1,\cdot \cdot \cdot \cdot \cdot \cdot ,n-1]\right)$, if s(x) is a cubic polynomial, the cubic polynomial formula is as follows:

(6) $$\begin{array}{c} s\left(x\right)=\left\{\begin{array}{c} s_{0}\left(x\right)x\in \left[x_{0},x_{1}\right]\\ s_{1}\left(x\right)x\in \left[x_{1},x_{2}\right]\\ \cdot \cdot \cdot \cdot \cdot \cdot \\ s_{n-1}\left(x\right)x\in \left[x_{n-1},x_{n}\right] \end{array}\right. \end{array}$$

(7) $$\begin{array}{c} s_{i}\left(x\right)=a_{i+1}\cdot x^{3}+b_{i+1}\cdot x^{2}+c_{i+1}\cdot x+d_{i+1} \end{array}$$

where $s_{i}\left(x\right)$ is the segmented cubic polynomial function for the cubic spline interpolation function s(x), and $\left(a_{0},a_{1},\cdots ,a_{n}\right)$, $\left(b_{0},b_{1},\cdots ,b_{n}\right)$, $\left(c_{0},c_{1},\cdots ,c_{n}\right)$, and $\left(d_{0},d_{1},\cdots ,d_{n}\right)$ are polynomial coefficients for the segmented cubic polynomial function $s_{0}\left(x\right),\cdots ,s_{n}\left(x\right)$.

Secondly, s(x) is a second-order continuous and differentiable within interval Δ.

## 3. Gene Correction Method for ARP

### 3.1. Basic Concepts and Application Principles of Gene Correction

Gene correction is a method of repairing problematic genes in a diseased individual so that they can be correctly expressed [14]. The appropriate use of gene correction is beneficial for enhancing the preservation effect of optimization, but if used excessively, it will lead to too many adjustable parameters, which will increase the amount of calculation required [15]. Considering computational efficiency, this paper applies genetic modifications to three situations generated in the optimization of the reference trajectory for parking: collision avoidance on the far side line of the parking garage, berthing inclination, and parking dislocation. The other situations are replaced with new boundary-crossing solutions for reverse parking.

### 3.2. Gene Correction for Collision on the Far Side Line of the Parking Garage

In the standard parking coordinate system, the far side line of the garage is equal to the vertical axis (y-axis). To solve the problem of collision on the far side line of parking garage, it is necessary to rotate the coordinate system counterclockwise based on the center points of the collected vehicle coverage areas within a certain range. Whether or not it is necessary to rotate the center point of the is-th collected vehicle coverage area counterclockwise, the empirical determination formula is as follows:

(8) $$\begin{array}{c} \left|y\left(P_{is}\right)-y\left(tPget\left({x_{lb,}}_{IT},{y_{lb,}}_{IT}\right)\right)\right|\leq \Delta Y_{fc\max} \end{array}$$

where $tPget\left({x_{lb,}}_{IT},{y_{lb,}}_{IT}\right)$ is the function for the bottom left corner $\left({x_{lb,}}_{IT},{y_{lb,}}_{IT}\right)$, and $\Delta Y_{fc\max}$ is the maximum ordinate offset.

The empirical estimation formula for correcting the abscissa movement $\Delta x_{is,fc}$ for collision avoidance on the far side line of parking garage is as follows:

(9) $$\begin{array}{c} \begin{array}{c} \Delta x_{is,fc}=\left(x\left(tPget\left({x_{lb,}}_{IT},{y_{lb,}}_{IT}\right)\right)-x\left(P_{is}\right)\right)\\ \;\;\;\;\;\;\;\;\;\;\;\;\;\;\;\;\;\;\;\;\;\;\;\;\;\;\;\;\;\;\;\;\;\;\cdot c_{fc}\cdot {x_{lb,}}_{IT} \end{array} \end{array}$$

where $c_{fc}$ is the empirical coefficient of collision avoidance on the far side line of the parking garage.

**Figure 2:**
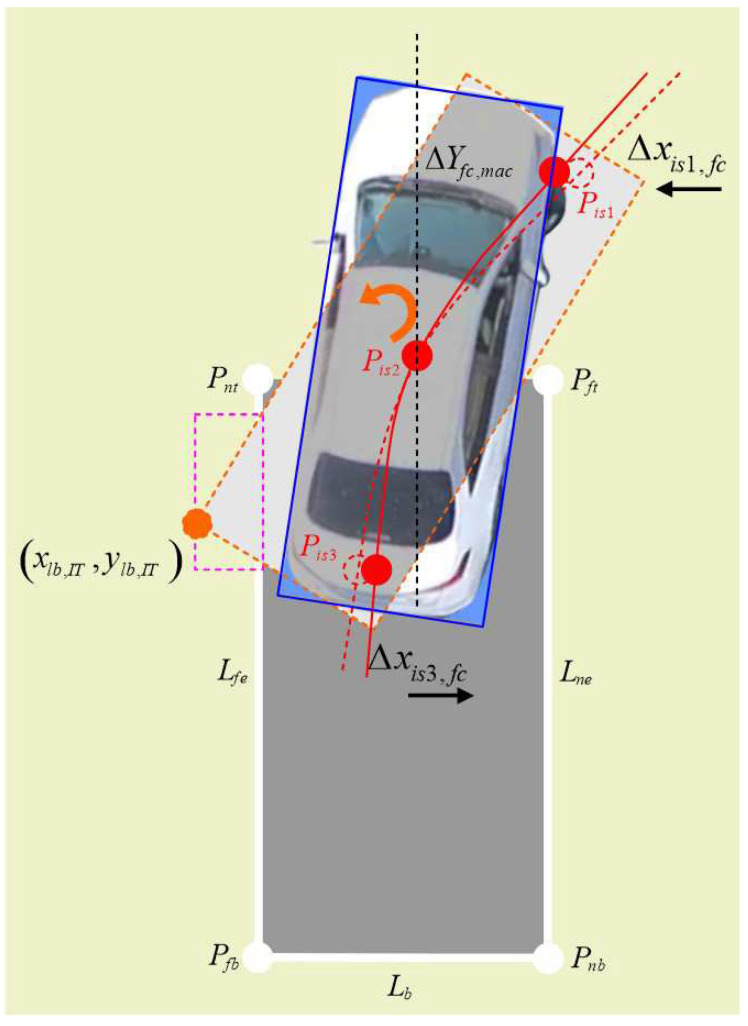
Schematic diagram of gene correction for collision avoidance on the far side line of the parking garage.

A schematic diagram of gene correction for collision avoidance on the far side line of the parking garage is shown in Figure 2.

In Figure 2, $P_{is2},=\left(x_{fc},y_{fc}\right)$, where $P_{is2}$ is the center point of the vehicle about the crossing boundary point for collision on the far side line of garage during the parking process.

### 3.3. Berthing Inclination Gene Correction

If the condition stating that the absolute value of the berthing inclination angle $\Delta {∠}_{S}$ must be less than the threshold value $\Delta {∠}_{S\max}$ is not met, it is called berthing inclination. If berthing inclination is to be corrected, the abscissa of some of the center points of the collected vehicle coverage areas should be moved slightly to the left. The empirical judgment formula for this left shift is as follows:

(10) $$\begin{array}{c} y\left(P_{is}\right)-y\left(P_{ns}\right)\leq \Delta Y_{SI\max}\; \end{array}$$

where $\Delta Y_{SI\max}$ is the maximum ordinate offset for correcting the berthing inclination.

The empirical estimation formula for obtaining the left-shift value $\Delta x_{is,SI}$ is as follows:

(11) $$\begin{array}{c} \Delta x_{is,SI}=\left(y\left(P_{is}\right)-y\left(P_{ns}\right)\right)\cdot \tan\left(\Delta {∠}_{S}-r\cdot \Delta {∠}_{S\max}\right) \end{array}$$

**Figure 3:**
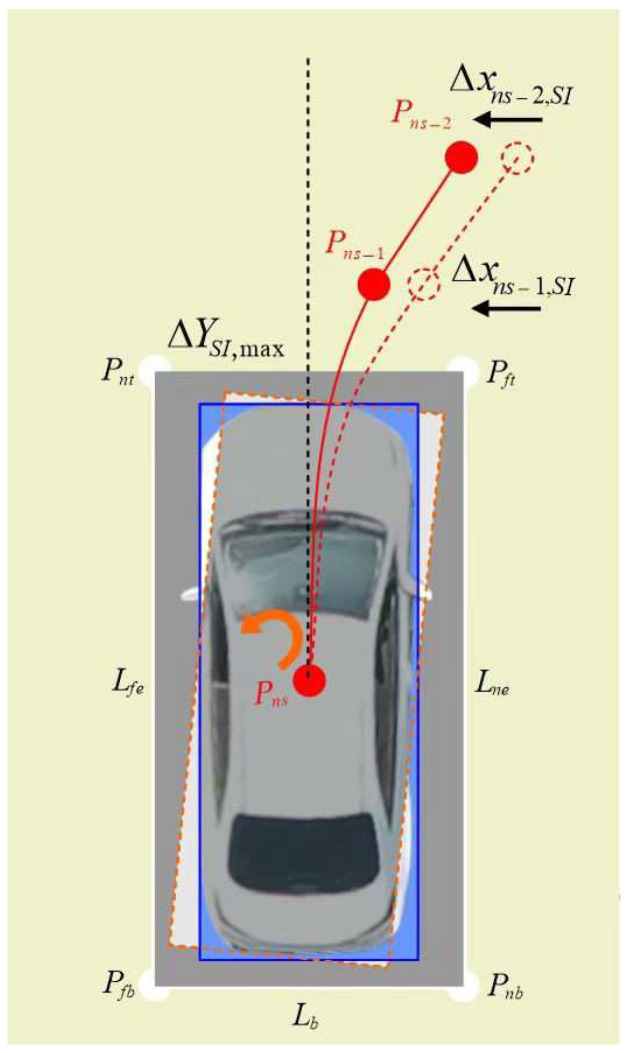
Schematic diagram of gene correction for berthing inclination.

A schematic diagram of gene correction for berthing inclination is shown in Figure 3.

In Figure 3, the abscissas of center points $P_{ns-1}$ and $P_{ns-2}$ of the collected vehicle coverage areas within the interval $\left(y_{ns},y_{ns}+\Delta Y_{SI\max}\right)$ are shifted to the left by $\Delta x_{ns-1,SI}$ and $\Delta x_{ns-2,SI}$, achieving the correction of berthing inclination.

### 3.4. Berthing Dislocation Gene Correction

If the condition stating that the absolute value of the berthing stop position error $\Delta y_{ns}$ must be less than the threshold value is not met, it is called berthing dislocation. In order to correct berthing dislocation, the ordinate of the center point of the parking area should be appropriately moved. The empirical estimation formula for the ordinate movement $\Delta y_{SP}$ of gene correction for berthing dislocation is as follows:

(12) $$\begin{array}{c} \Delta y_{SP}=\left|\Delta y_{ns}\right|-r\cdot \Delta {y_{H}}_{\max} \end{array}$$

**Figure 4:**
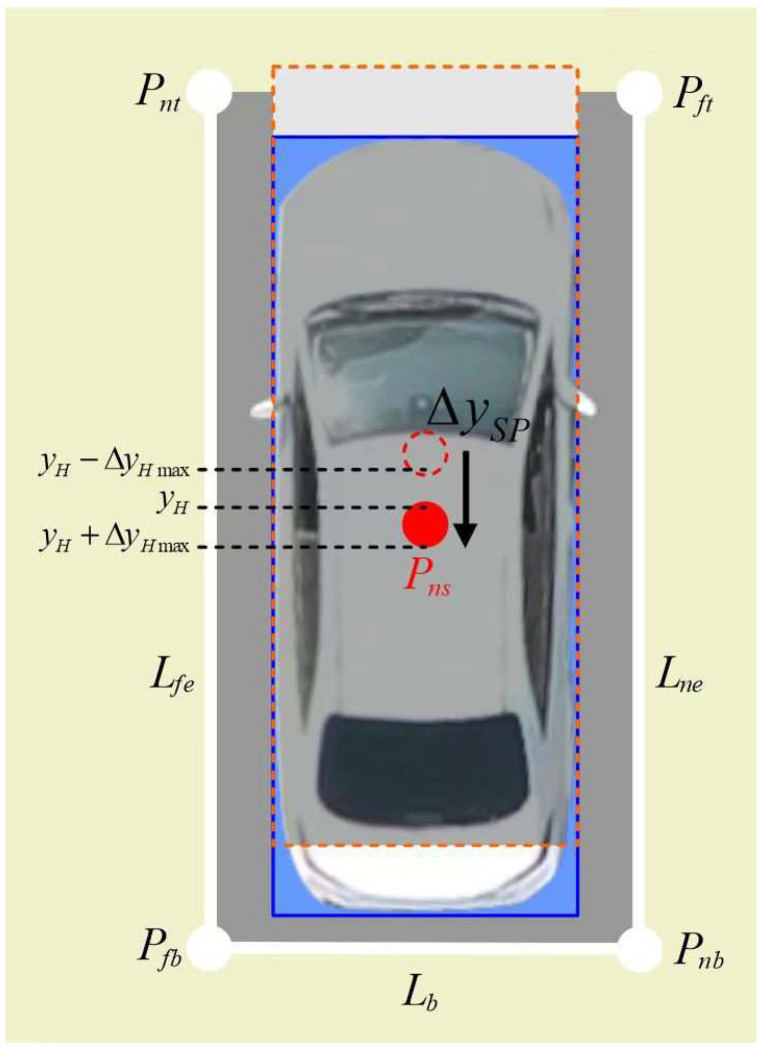
Schematic diagram of gene correction for berthing dislocation.

A schematic diagram of gene correction for berthing dislocation is shown in Figure 4.

In Figure 4, since the center point $P_{ns}$ of the parking area is not in the allowable parking range $\left(y_{H}-\Delta {y_{H}}_{\max},y_{H}+\Delta {y_{H}}_{\max}\right)$, it is above the parking area and is moved down to $\Delta y_{SP}$ to achieve the goal of correcting berthing dislocation.

Based on the gene correction method proposed in this study, only three empirical parameters need to be added: the maximum ordinate offset $\Delta Y_{fc\max}$ and the empirical coefficient $c_{fc}$ of abscissa movement for correcting collision on the far side line of the parking garage, and the maximum ordinate offset $\Delta Y_{SI\max}$ for correcting berthing inclination.

## 4. Improved Immune Moth–Flame Optimization

### 4.1. Moth–Flame Optimization

Moth–flame optimization (MFO), proposed in 2015, imitates the behavior of moths searching for a light source through lateral positioning [16]. The optimization process of MFO can be abstractly represented by a triplet [17]. The mapping expressions are as follows:

(13) $$\begin{array}{c} MFO=\left(I,P,T\right) \end{array}$$

(14) $$\begin{array}{c} I:\phi \to \left\{M,OM,F,OF\right\} \end{array}$$

(15) $$\begin{array}{c} P:M\to M \end{array}$$

(16) $$\begin{array}{c} T:\left\{\begin{array}{c} M_{i}=\omega \cdot D_{ij}\cdot e^{\tau t}\cdot \cos\left(2\pi t\right)+F_{j}\\ D_{ij}=\left|M_{i}-F_{j}\right| \end{array}\right. \end{array}$$

where *I* represents initializing the moth population and flame set and calculating the fitness value of each moth and flame, *M* is the moth population, OM is its fitness value vector matrix, *F* is the flame set, OF is its fitness value vector matrix, $M=\left[\begin{array}{cccc} M_{11} & M_{12} & \cdots & M_{1d}\\ M_{21} & M_{22} & \cdots & M_{2d}\\ \vdots & \vdots & \vdots & \vdots \\ M_{n1} & M_{n2} & \cdots & M_{nd} \end{array}\right]$, $OM=\left[\begin{array}{c} OM_{1}\\ OM_{2}\\ \vdots \\ OM_{n} \end{array}\right]$, $F=\left[\begin{array}{cccc} F_{11} & F_{12} & \cdots & F_{1d}\\ F_{21} & F_{22} & \cdots & F_{2d}\\ \vdots & \vdots & \vdots & \vdots \\ F_{n_{f}1} & F_{n_{f}2} & \cdots & F_{n_{f}d} \end{array}\right]$, $OF=\left[\begin{array}{c} OF_{1}\\ OF_{2}\\ \vdots \\ OF_{n_{f}} \end{array}\right]$, *n* is the population size, *d* is the solution dimension, and $n_{f}$ is the number of flames (at the beginning, the number of flames is equal to the population size), $n_{f}=n$; *P* represents updating the running trajectory of the moth population (after updating the moth population, the fitness value of each individual moth should be recalculated; if the fitness value is better than the current flame, the flame set will be updated), *T* represents updating position of moths according to the logarithmic spiral law, $D_{ij}\cdot e^{\tau t}\cdot \cos\left(2\pi t\right)$ is the logarithmic spiral term, $M_{i}$ is the position of the *i*-th moth, $F_{j}$ is the position of the *j*-th flame, $D_{ij}$ is the Euclidean distance between the *i*-th moth and the *j*-th flame, τ is the logarithmic spiral shape adjustment coefficient, *t* is a random number in the interval $\left(-1,1\right)$, and ω is the weight coefficient.

The number of flames will be reduced gradually during the iteration [18]. The formula for updating the number of flames is as follows:

(17) $$\begin{array}{c} n_{f}=round\left(n-t\frac{n-1}{t_{\max}}\right) \end{array}$$

where *t* is the current iteration number, $t_{\max}$ is the maximum number of iterations, and round(x) represents *x* rounded to the nearest integer.

### 4.2. Immune Mechanism

In this study, the concentration selection mechanism in the artificial immune algorithm for adjusting the distribution of moths in space is introduced into the MFO to improve its global optimization performance [19]. In the concentration selection mechanism, the formulas for calculating antibody concentration and concentration probability are as follows:

(18) $$\begin{array}{c} D\left(x_{i}\right)=\frac{1}{\sum_{j=1}^{m+N}\left|f\left(x_{i}\right)-f\left(x_{j}\right)\right|} \end{array}$$

(19) $$\begin{array}{c} P_{d}\left(x_{i}\right)=\frac{\frac{1}{D\left(x_{i}\right)}}{\sum_{i=1}^{m+N}\frac{1}{D\left(x_{i}\right)}}=\frac{\sum_{j=1}^{m+N}\left|f\left(x_{i}\right)-f\left(x_{j}\right)\right|}{\sum_{i=1}^{m+N}\sum_{j=1}^{m+N}\left|f\left(x_{i}\right)-f\left(x_{j}\right)\right|} \end{array}$$

where $f\left(x_{i}\right)$, $D\left(x_{i}\right)$, and $P_{d}\left(x_{i}\right)$ are the fitness function value, antibody concentration, and concentration probability of the *i*-th moth, respectively, and i=1,2,⋯,m+N.

### 4.3. Nonlinear Decline Strategy of Weight Coefficient

If the weight coefficient ω is large, the swarm intelligence optimization algorithm offers strong global exploration ability and is suitable for the early stage of the optimization process; contrarily, if the weight coefficient is small, the algorithm’s local development ability is strong and it is suitable for the later stage [20]. Several nonlinear decline strategies for weight coefficients ω with strong plasticity are conducive improving the global optimization ability of MFO [21]. The two weight coefficient calculation formulas ω are as follows:

(20) $$\begin{array}{c} \omega =\omega_{\min}+\omega_{d}\cdot \cos{\left(\left(1-t_{r}\right)\cdot \frac{\pi }{2}\right)}^{\beta } \end{array}$$

where $\omega_{d}$ is the decreasing weight coefficient; $\omega_{d}=\omega_{m\mathrm{ax}}-\omega_{\min}$, $\omega_{\min}$, and $\omega_{\max}$ are the minimum and maximum weight coefficient values, respectively; $t_{r}$ is the iteration progress; $t_{r}=t\cdot {t_{\max}}^{-1}$; and β is the optimization factor.

(21) $$\begin{array}{c} \omega =\omega_{\max}-\omega_{d}*{\left(\frac{l}{N_{\mathrm{int}}^{\max}}\right)}^{\alpha } \end{array}$$

where α is the decline rate and exhibits a significant difference throughout the whole iteration process.

According to the above formulas, the nonlinear declining trend for the weight coefficient ω can be optimized and adjusted. The two calculation formulas above have excellent effects on improving global optimization ability; in different scenarios the improvement effects are discrepant, and to choose the best option, extensive tests should be carried out.

### 4.4. Nonlinear Decline Strategy for Crossover and Mutation Probability

In the early stage of iterative optimization, population diversity should be emphasized, the intensity of gene change should be enhanced, and global exploration should be strengthened to improve individual adaptability. Following iteration optimization, preserving the existing optimization results should be emphasized, the intensity of gene changes should be reduced, and local development should be strengthened to avoid the destruction of existing advantageous genes. Therefore, mutation and crossover probability should decrease gradually with an increasing number of iterations. This paper presents a nonlinear decline strategy for directional variation and crossover probabilities $p_{c}$ and $p_{m}$ with strong smoothness based on exponential formal decline. The formulas for calculating mutation and crossover probabilities $p_{c}$ and $p_{m}$ are as follows:

(22) $$\begin{array}{c} p_{c}={p_{c,}}_{\min}+{p_{c,}}_{\max}\cdot e^{{t_{r}}^{\beta_{c}}\cdot \ln\left(\frac{{p_{c,}}_{\min}}{{p_{c,}}_{\max}}\right)} \end{array}$$

(23) $$\begin{array}{c} p_{m}={p_{m,}}_{\min}+{p_{m,}}_{\max}\cdot e^{{t_{r}}^{\beta_{m}}\cdot \ln\left(\frac{{p_{m,}}_{\min}}{{p_{m,}}_{\max}}\right)} \end{array}$$

where ${p_{c,}}_{\min}$, ${p_{c,}}_{\max}$, ${p_{m,}}_{\min}$, and ${p_{m,}}_{\max}$ are the minimum and maximum crossover probability and the minimum and maximum mutation probability, respectively, and $\beta_{m}$ and $\beta_{c}$ are the the optimization factors for the nonlinear decline of mutation and crossover probabilities $p_{m}$ and $p_{c}$, respectively.

According to the above formulas, the exponential form of the nonlinear decline strategy is adopted, and the nonlinear decline trend can be optimized and adjusted by selecting the most suitable optimization factors, $\beta_{m}$ and $\beta_{c}$. This strategy has strong smoothness, and its nonlinear decline is particularly smooth. This strategy can adjust the decline rates of mutation and cross probability in real time during iteration, which is conducive to improving the balance between global exploration and local exploitation, and thus, improving the global optimization ability of the algorithm.

### 4.5. High-Quality Solution-Set Maintenance Mechanism Based on Fusion Distance

In the evolution process of each iteration, a high-quality solution set should be established to preserve the existing optimization achievements. To prevent adverse effects on the algorithm’s computational efficiency, the high-quality solution-set size $HS_{s}$ should be less than the allowable maximum $HS_{AS}$, that is, $HS_{S}\leq HS_{AS}$. So, the design of an elite set maintenance mechanism is very significant for MFO. In terms of the definition of the Mahalanobis distance $M_{d}$, the $M_{d}$ results between two samples under identical states in different reference spaces will differ because of the difference in their correlation of variables. Since the Euclidean distance $E_{d}$ considers the independence of characteristic variables, and $M_{d}$ considers the independence of characteristic variables, this study combines $M_{d}$ and $E_{d}$ to effectively measure distance in the case of fuzzy correlation, and a new calculation method called “Fusion distance $F_{d}$” that considers the correlation and independence between feature variables is given [22]. The “Fusion distance $F_{d}$” equation is as follows:

(24) $$\begin{array}{c} F_{d}=\mu \cdot M_{d}\left(Y_{b},X_{a}\right)+\left(1-\mu \right)\cdot E_{d}\left(Y_{b},X_{a}\right) \end{array}$$

(25) $$\begin{array}{c} \mu =\sqrt{1-\left|CM_{ba}\right|} \end{array}$$

(26) $$\begin{array}{c} CM_{ba}=\left[\begin{array}{c} \begin{array}{c} \rho \left(y_{1},x_{1}\right) \end{array}\rho \left(y_{1},x_{2}\right)\cdots \rho \left(y_{1},x_{a}\right)\\ \begin{array}{c} \rho \left(y_{2},x_{1}\right) \end{array}\rho \left(y_{2},x_{2}\right)\cdots \rho \left(y_{2},x_{a}\right)\\ \vdots \;\;\vdots \;\ddots \vdots \\ \begin{array}{c} \rho \left(y_{b},x_{1}\right) \end{array}\rho \left(y_{b},x_{2}\right)\cdots \rho \left(y_{b},x_{a}\right) \end{array}\right] \end{array}$$

where $Y_{b}$ and $Y_{a}$ denote the reference vectors with *b* and *a* elements to be tested; $M_{d}\left(Y_{b},X_{a}\right)$ denotes the Mahalanobis distance between vectors $Y_{b}$ and $X_{a}$; $E_{d}\left(Y_{b},X_{a}\right)$ denotes the Ed between vectors $Y_{b}$ and $X_{a}$; $CM_{ba}$ denotes the correlation coefficient matrix between vectors $Y_{b}$ and $X_{a}$; and μ represents the parameters used to ensure that the sum is meaningful and the distance adjustment is on the same scale.

Due to the introduction of the correlation coefficient calculation in μ, μ reflects the adaptability of distance and the additivity on the distance scale [23].

## 5. ARP Experiment

### 5.1. Description of Experimental ARP Scene

In this study, we chose automatic parking garage No. 160 of the Dalian Shell Museum in Dalian, Xinghai Square, for the parking experiment. The near side and far side lines of the experimental garage are equal in length at 5 m long, the bottom line of the garage is 2.5 m long, and the width of the marker line is 0.1 m. The experimental vehicle model was a Toyota Corolla 1.2T S-CVT GL Pioneer Version (2019 model), with a length of 4635 mm, a width of 1780 mm, and a height of 1455 mm. The horizontal and longitudinal distances from the bottom right corner of the starting area of the experimental vehicle to the near corner point at the top of the garage were 2 m and 2.1 m, respectively.

### 5.2. Design and Configuration of ARP Experiment

**Figure 5:**
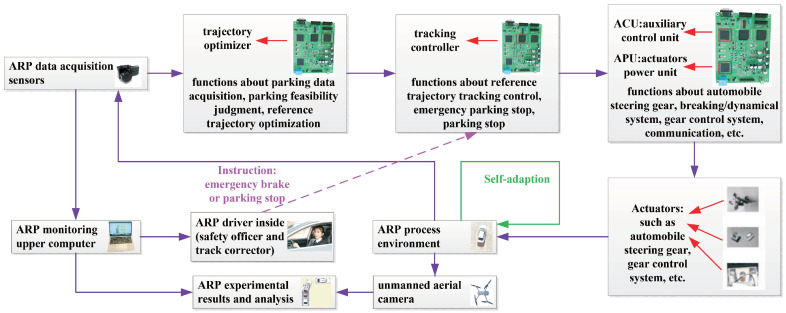
Diagram showing the design of the ARP experiment.

The experimental vehicle used in this study is equipped with an ARP system, which has a real parking data acquisition sensor, a parking feasibility judgment device, a reference trajectory optimizer, a parking controller, a parking stop device, an emergency brake device, a parking image system, and a parking monitoring upper computer. The onboard sensors are mainly used for parking feasibility judgment and reference trajectory tracking control. In the parking process, the reference trajectory optimizer transmits the optimized reference trajectory to the parking controller and the upper parking monitoring computer, which not only provides the necessary reference trajectory for tracking control, but also enables safe parking, as the driver has a more comprehensive view of the real-time parking conditions. The ARP system exhibits powerful performance, especially regarding safety; once an accident risk is perceived, the emergency brake will be activated immediately. However, research on ARP is still in its early stages; security is critically important for this technology, and there is no room for error. The ARP experiment in this study was closely supervised by safety assurance personnel; the driver had a C2 driver’s license and acted as both a safety officer and an enhanced track control corrector, the ground safety inspector was able to contact the driver, and we employed an aerial drone photographer with good photography skills. A diagram of the design of the ARP experiment is shown in Figure 5.

As can be seen from Figure 5, in the ARP process, the ARP driver did not interfere with the normal parking tracking control, and would only activate the experimental vehicle’s stop or emergency brake function if necessary; some of the experimental results were directly obtained from the ARP process environment using an unmanned aerial camera, and some were indirectly obtained using ARP data acquisition sensors and an upper ARP monitoring computer.

The main parameters of the automatic parking experiment were as follows: The parking data acquisition, parking feasibility judgment, reference trajectory optimization, reference trajectory tracking control, emergency parking stop, and parking stop limit times were 3.5 s, 1.5 s, 12 s, 25 s, 0.4 s, and 1.5 s, respectively. One unmanned aerial camera (Mavic 2 DJI’s, ’Yu’, from Dajiang, Shenzhen, China) was used, and the upper computer for parking monitoring was a ’MacBook Pro 2016 Core i5 @ 2.9 GHZ’, which used Bluetooth for vehicle communication and was fixed to the mobile phone holder of the vehicle. Two MPC555LFMZP40 chips were used for the trajectory optimizer (used for parking data acquisition, parking feasibility judgment, and reference trajectory optimization) and tracking controller (used for reference trajectory tracking control, emergency parking stop, and parking stop), and two DSP28335 chips were used for the auxiliary control unit (ACU, used for the air-conditioner, overload protection device, communication, etc.) and actuator power unit (APU, used for the breaker, automobile steering gear, breaking/dynamical system, gear control system, etc.).

### 5.3. Experimental Results and Analysis of ARP

In order to verify the effectiveness of the optimization algorithm proposed in this study based on a real ARP scene in clear and windless weather, the IIMFO-GC proposed in this study, the IIMFO proposed in [13], the levy flight-based moth–flame optimization method proposed in [24], the MFO proposed in [25], the IPSO proposed in [4], and the PSO proposed in [26] were adopted to implement ARP reference trajectory optimization. Fuzzy proportional–integral–derivative (Fuzzy PID) control was adopted to implement parking tracking control for the reference trajectory. In order to achieve a fair comparison between the six algorithms, the same parameters were used for the optimization model as for the ARP reference trajectory, and are shown in Table 1. The parameters of IIMFO-GC for the ARP reference trajectory are shown in Table 2.

**Figure 6:**
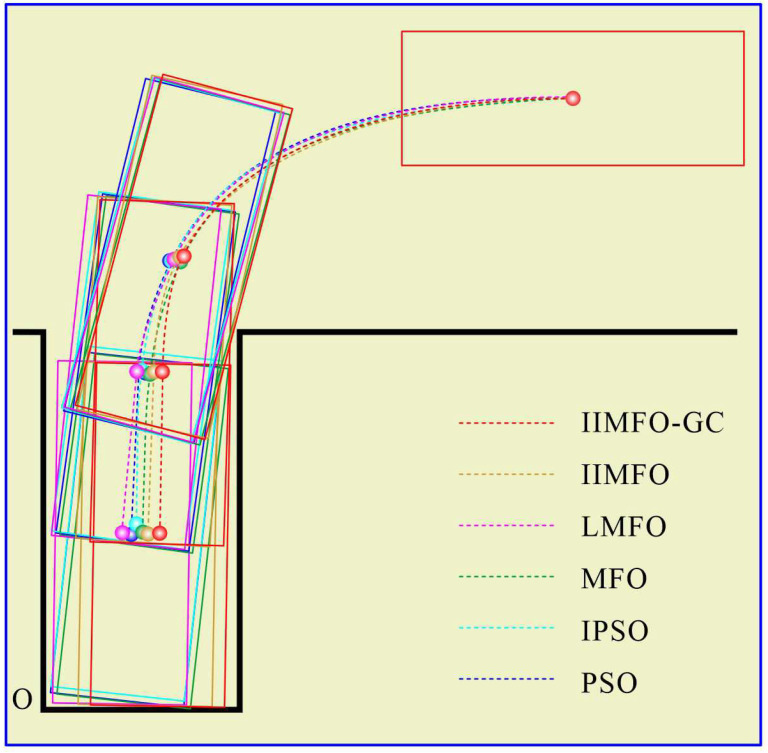
Results obtained using parking monitoring host computer for optimization of fixed points of collected vehicle coverage areas.

**Figure 7:**
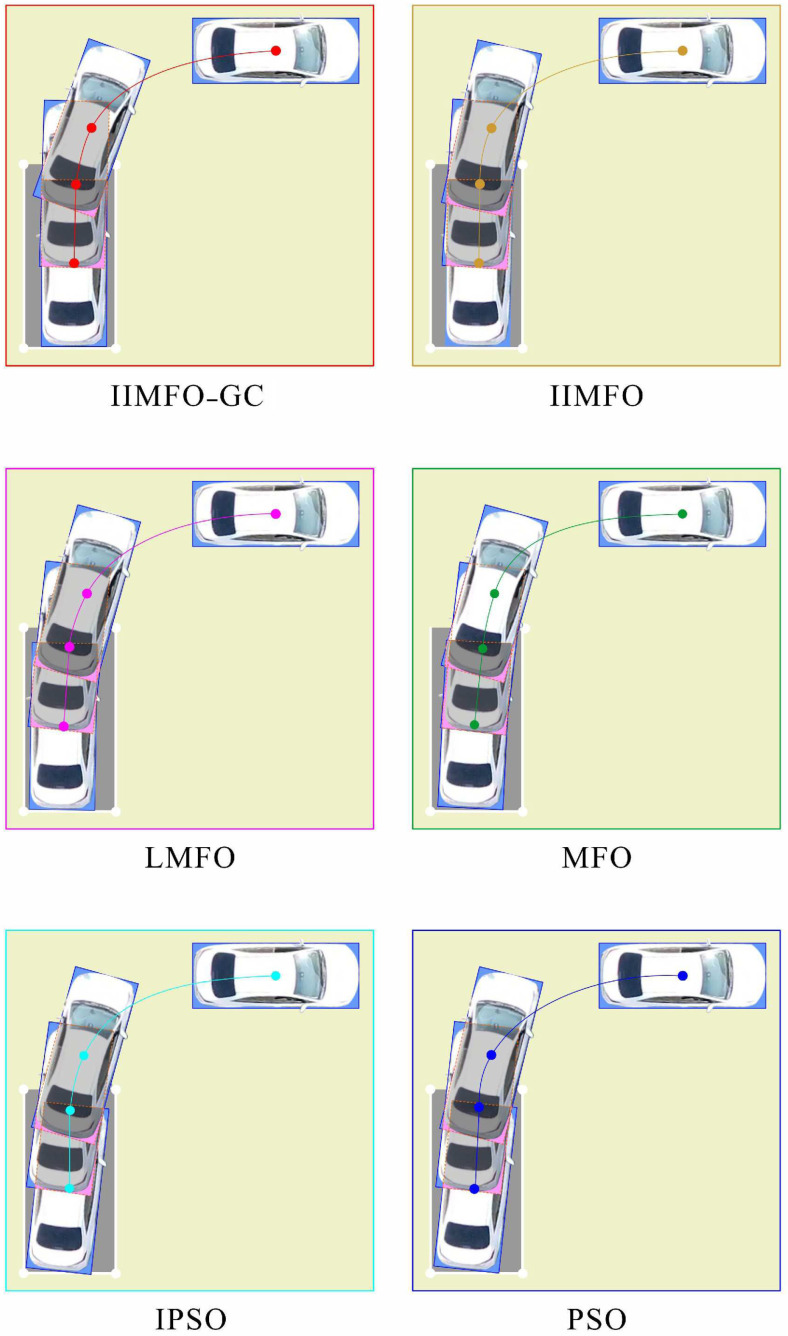
Results obtained using the parking monitoring host computer for tracking control of fixed points of collected vehicle coverage areas.

**Figure 8:**
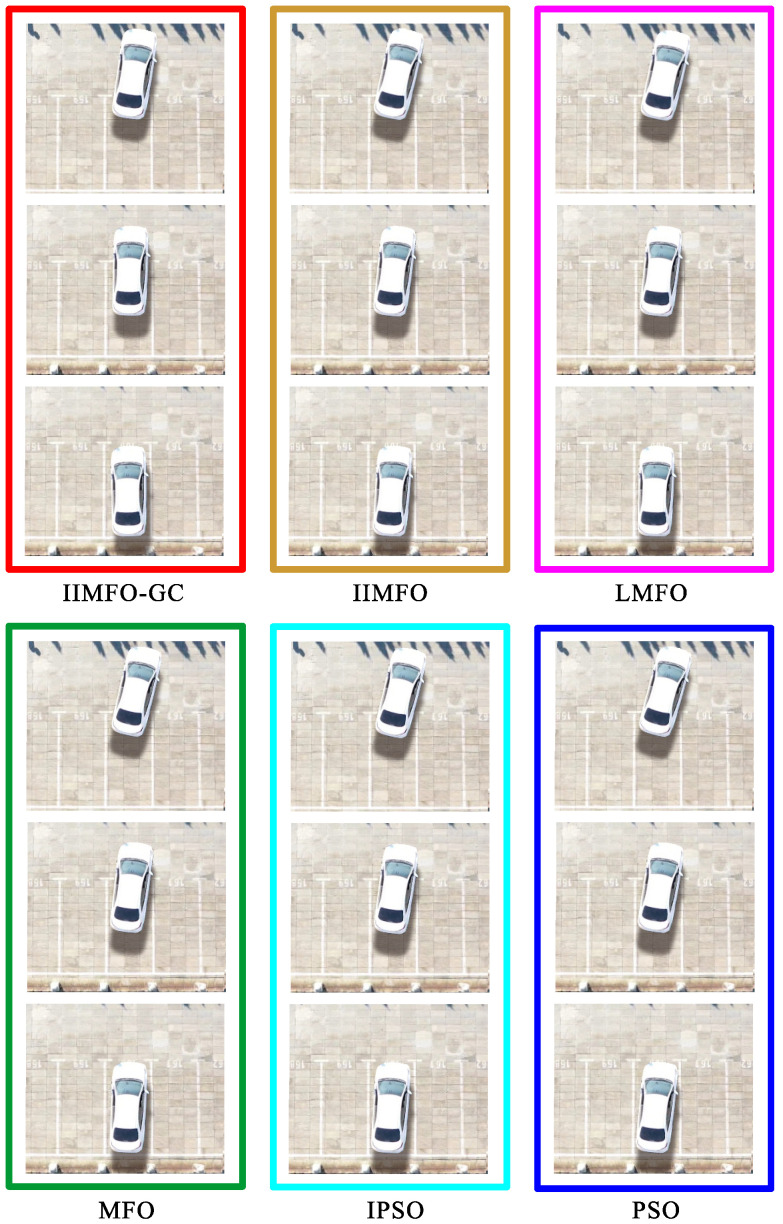
Unmanned aerial images of actual scene for fixed points of collected vehicle coverage areas.

**Figure 9:**
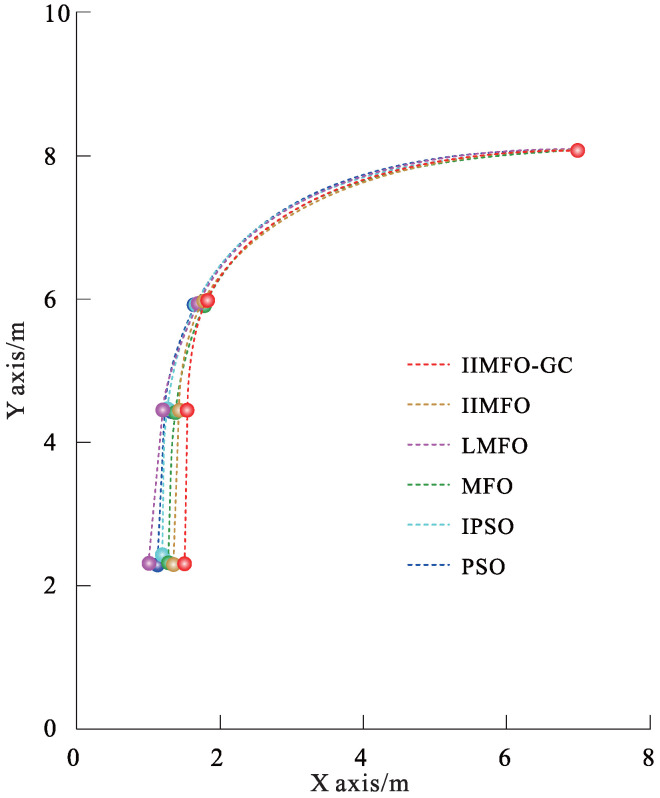
Optimization curves for the reference trajectories of ARP.

**Figure 10:**
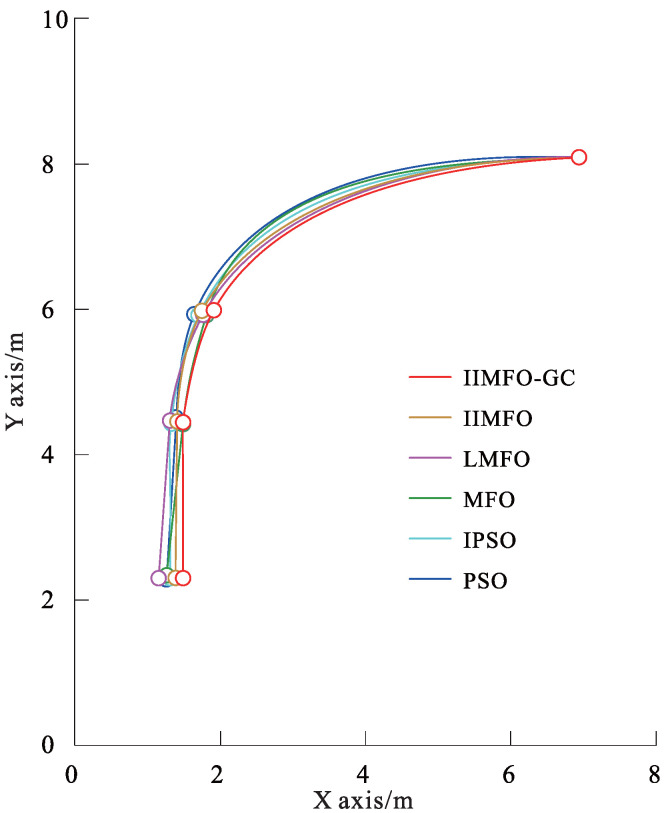
Tracking control curves for the reference trajectories of ARP.

**Figure 11:**
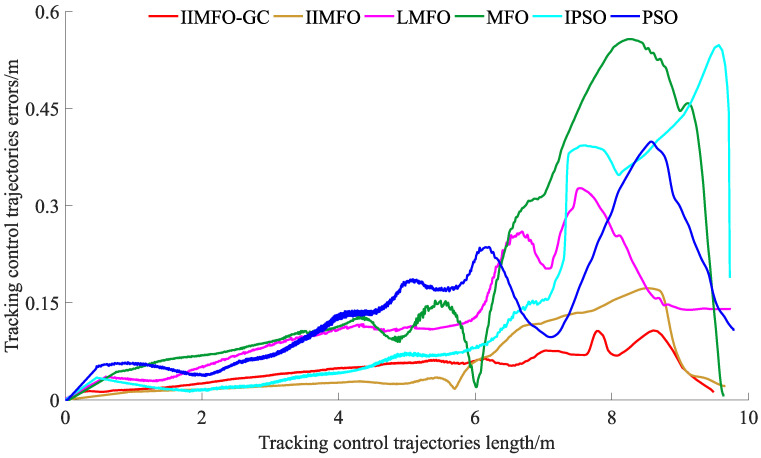
Tracking control trajectory error curves of whole ARP process.

The experimental results for actual reverse parking given in this study include not only the optimization and tracking control results obtained using the parking monitoring host computer, but also the unmanned aerial photography images of several fixed points of the collected vehicle coverage areas during reverse parking. In order to achieve a fair comparison between the six algorithms, the same three fixed points of the collected vehicle coverage areas were selected: parking point $P_{4}$ followed by $y\left(P_{4}\right)\in \left[5.95,6.05\right]$ m, parking point $P_{7}$ followed by $y\left(P_{7}\right)\in \left[4.45,4.55\right]$ m, and parking stop point $P_{10}$ followed by $y\left(P_{10}\right)\in \left[2.30,2.50\right]$ m. The results obtained using the parking monitoring host computer for optimization and tracking control at fixed points of the collected vehicle coverage areas are shown in Figure 6 and Figure 7; the unmanned aerial images of the actual scene at fixed points of the collected vehicle coverage areas are shown in Figure 8; optimization and tracking control curves for the reference trajectories of automatic reverse parking are shown in Figure 9 and Figure 10; and tracking control trajectory error curves of the whole ARP process are shown in Figure 11. The position, path length, and berthing inclination angle results for automatic reverse parking optimization and tracking control are shown in Table 3 and Table 4. The tracking control errors in the position, path length, and berthing inclination angle results are shown in Table 5. The computational costs of each algorithm are shown in Table 6.

As can be seen from Table 3 and Figure 9, compared with the other five optimization algorithms, the IIMFO-GC proposed in this study exhibits a better reference trajectory; in particular, as shown in Figure 9, the reference trajectory path of IIMFO-GC is shorter. As can be seen from Table 4 and Figure 10, compared with the other five optimization algorithms, based on the same tracking control algorithm (Fuzzy PID), the IIMFO-GC proposed in this study obtains better tracking control results; in particular, as shown in Figure 10, the actual parking path of IIMFO-GC is shorter. As can be seen from Table 5 and Figure 11, compared with the other five optimization algorithms, based on the same tracking control algorithm (Fuzzy PID), the IIMFO-GC proposed in this study obtains tracking control results with fewer errors; in particular, as shown in Figure 11, the error in the tracking control of IIMFO-GC is smaller, and its tracking control trajectory errors for whole ARP process are fewer. Figure 7 shows that compared with the other five optimization algorithms, the IIMFO-GC proposed in this study exhibits a better reference trajectory; moreover, the optimized reference trajectory is closer to the near side line of parking garage and has a smaller expected berth inclination angle. As can be seen from Table 5, compared with the other five optimization algorithms, the IIMFO-GC proposed in this study has a better berthing inclination angle for both optimization and tracking control. Table 6 and Figure 6 and Figure 8 show that compared with the other five optimization algorithms, based on the same tracking control algorithm (Fuzzy PID), the IIMFO-GC proposed in this study obtains a better tracking control path; moreover, the tracking control path is closer to the near side line of the parking garage, and the actual berth inclination angle is smaller. As shown in Table 7, compared with the other five optimization algorithms, the IIMFO-GC proposed in this study is completed in a shorter time.

## 6. Conclusions

This paper proposes an IIMFO-GC for intelligent ARP function. Specifically, a novel reference trajectory optimization model for ARP is constructed, and a conversion principle for the ARP plane coordinate system is introduced; considering both the reference trajectory length and berthing inclination angle, a gene correction method for ARP is proposed for improving reference trajectory optimization performance. The proposed IIMFO-GC for the ARP trajectory optimizer effectively improves optimization performance.

The main contributions of this paper are as follows:

(I) Improvements to the ARP trajectory optimizer: The traditional reference trajectory optimization model is not suitable, and it is diffucult to establish effective boundary-crossing solutions for reverse parking. Therefore, in this study, based on the standard automatic parking plane system, a reasonable high-quality reference trajectory optimization model for ARP is constructed by combining the cubic spline-fitting method and a highly effective gene correction method.

(II) Improvements to the MFO algorithm: this paper proposes using IIMFO-GC to obtain a more appropriate reference trajectory for ARP; the following improvements are proposed: (II) integrate the immune mechanism with a high-quality solution-set maintenance mechanism based on fusion distance to strengthen global optimization quality; (I) introduce nonlinear decline strategies for weight coefficients and crossover and mutation probabilities to further strengthen global optimization ability.

These experimental results indicate that using IIMFO-GC for intelligent ARP function provides a better reference trajectory optimization effect than traditional optimization algorithms. Further, the comparison of the experimental results based on intelligent ARP function indicates that IIMFO-GC has a better control effect than anticipated.

However, there are several shortcomings in this research:

(I) Sensor accuracy has certain effects on ARP control.

(II) The effectiveness of the strategies for integrating the immune mechanism with moth–flame optimization is finite.

(III) The ARP scene used for the experimental comparison represents an ideal scenario, so the effectiveness of our experimental comparison is limited and one-sided.

We propose several future research directions for the use of MFO for ARP:

(I) Conduct further research on precise ARP trajectory data (such as position, length, and inclination angle) and measuring algorithms.

(II) Investigate existing improved MFOs, such as EMFO or Lévy-fight MFO, to further improve MFO optimization performance [18,24].

(III) Enhance the MFO algorithm by integrating other mechanisms or methods (such as the dual-population genetic mechanism) to further improve optimization quality [27].

(IV) Use several (more than two) typical ARP scenes for experimental comparison to further improve verification precision [28].

## Figures and Tables

**Table 1:** Parameters of optimization model for ARP reference trajectory.

Name	Symbol	Value
Starting inclination angle threshold	$\Delta {∠}_{B\max}$	$\frac{\pi }{75}$
Vehicle coverage area collection times	ns	10
Parking inclination angle threshold	$\Delta {∠}_{S\max}$	$\frac{\pi }{20}$
Parking position error threshold	$\Delta y_{ns}$	0.15 m
Parking process period	Δt	1.5 × $10^{-3}$ s
Attitude angle difference threshold	$\Delta {∠}_{\max}$	5 × $10^{-4}\pi$
Parking interval threshold	$\Delta L_{\max}$	$10^{-3}$
Parking position expected ordinate	$y_{H}$	2.35 m

**Table 2:** Parameter configuration of IIMFO-GC for ARP reference trajectory.

Name	Symbol	Value
Moth population size	*n*	30
Iteration number	$t_{\max}$	80
Logarithmic spiral shape adjustment coefficient	τ	$\frac{\pi }{20}$
Minimum weight coefficient	$\omega_{\min}$	0.4
Maximum weight coefficient	$\omega_{\max}$	0.9
Weight coefficient optimization factor	β	1.85
Minimum crossover and mutation probabilities	${p_{c,}}_{\min}$,${p_{m,}}_{\min}$	0.4,0.1
Maximum crossover and mutation probabilities	${p_{c,}}_{\max}$,${p_{m,}}_{\max}$	0.8,0.3
Crossover probability optimization factor	$\beta_{m}$	1.66
Mutation probability optimization factor	$\beta_{c}$	1.49
Collision correction maximum ordinate offset	$\Delta Y_{fc\max}$	0.92 m
Collision correction empirical coefficient	$c_{fc}$	0.58
Berthing inclination maximum ordinate offset	$y_{H}$	2.35 m

**Table 3:** Position and path length results of reference trajectory optimization for ARP.

Algorithms	Coordinates of $p_{10}$, $p_{7}$, $p_{4}$ (m)	Path Length (m)
IIMFO-GC	(1.52,2.29), (1.55,4.43), (1.84,5.97)	9.480
IIMFO	(1.37,2.29), (1.44,4.43), (1.78,5.96)	9.659
LMFO	(1.02,2.30), (1.20,4.44), (1.69,5.91)	9.694
MFO	(1.29,2.31), (1.37,4.48), (1.80,5.87)	9.619
IPSO	(1.21,2.43), (1.29,4.46), (1.64,5.90)	9.732
PSO	(1.14,2.27), (1.32,4.40), (1.61,5.91)	9.829

**Table 4:** Position and path length results of reference trajectory tracking control for ARP.

Algorithms	Coordinates of $p_{10}$, $p_{7}$, $p_{4}$ (m)	Path Length (m)
IIMFO-GC	(1.51,2.29), (1.50,4.42), (1.90,5.96)	9.493
IIMFO	(1.39,2.30), (1.38,4.43), (1.74,5.95)	9.658
LMFO	(1.16,2.29), (1.30,4.45), (1.80,5.89)	9.741
MFO	(1.28,2.31), (1.52,4.39), (1.84,5.88)	9.635
IPSO	(1.31,2.27), (1.34,4.42), (1.70,5.88)	9.728
PSO	(1.27,2.25), (1.40,4.50), (1.66,5.91)	9.785

**Table 5:** Berthing inclination angle results of reference trajectory optimization and tracking control for ARP.

Algorithms	Optimization	Tracking Control
IIMFO-GC	0.0169	0.0177
IIMFO	0.0190	0.0204
LMFO	0.0181	0.0193
MFO	0.1113	0.0530
PSO	0.1093	0.1085
PSO	0.1098	0.1078

**Table 6:** Tracking control errors in position, path length, and berthing inclination angle results for ARP.

Algorithms	Coordinates of $p_{10}$, $p_{7}$, $p_{4}$ (m)	Path Length (m)	Berthing Inclination Angle
IIMFO-GC	(0.01,0), (0.05,0.01), (0.06,0.01)	0.013	0.0008
IIMFO	(0.02,0.01), (0.06,0), (0.04,0.01)	0.001	0.0014
LMFO	(0.14,0.01), (0.10,0.01), (0.11,0.02)	0.047	0.0012
MFO	(0.01,0), (0.15,0.09), (0.04,0.01)	0.016	0.0583
IPSO	(0.10,0.16), (0.05,0.04), (0.06,0.02)	0.004	0.0008
PSO	(0.13,0.02), (0.08,0.10), (0.04,0)	0.044	0.0020

**Table 7:** Computational cost of each algorithm performed for ARP.

Algorithms	Computational Cost (s)
IIMFO-GC	9.335
IIMFO	9.754
LMFO	10.108
MFO	10.227
PSO	9.935
PSO	10.040

## Data Availability

Data are contained within the article.

## Abbreviations

The following abbreviations are used in this manuscript:

ARP	automatic reverse parking
IIMFO-GC	improved immune moth–flame optimization based on gene correction
MFO	moth–flame optimization
